# Formulation and Evaluation of a Drug-in-Adhesive Patch for Transdermal Delivery of Colchicine

**DOI:** 10.3390/pharmaceutics14102245

**Published:** 2022-10-21

**Authors:** Yaran Lei, Guobao Yang, Feng Du, Jiahe Yi, Liangzhu Quan, Hanhan Liu, Xun Zhou, Wei Gong, Jing Han, Yuli Wang, Chunsheng Gao

**Affiliations:** 1 School of Pharmaceutical Engineering, Shenyang Pharmaceutical University, Benxi 117004, China; 2 State Key Laboratory of Toxicology and Medical Countermeasures, Beijing Institute of Pharmacology and Toxicology, Beijing 100850, China; 3 School of Pharmacy, Guangxi Medical University, Nanning 530021, China; 4 College of Pharmacy, Henan University, Kaifeng 475000, China; 5 Faculty of Functional Food and Wine, Shenyang Pharmaceutical University, Shenyang 110016, China

**Keywords:** colchicine, gout, drug-in-adhesive, transdermal

## Abstract

Gout is one of the most prevalent rheumatic diseases, globally. Colchicine (COL) is the first-line drug used for the treatment of acute gout. However, the oral administration of COL is restricted, owing to serious adverse reactions. Therefore, this study aimed to develop a drug-in-adhesive (DIA) patch to achieve transdermal delivery of COL. We investigated the solubility of COL in different pressure-sensitive adhesives (PSAs) using slide crystallization studies. The COL-DIA patches were optimized based on in vitro skin penetration studies and evaluated by in vivo pharmacokinetics and pharmacodynamics. The results showed that the optimized COL-DIA patch contained 10% COL, Duro-Tak 87-2516 as PSA, 5% oleic acid (OA) and 5% propylene glycol (PG) as permeation enhancer, exhibiting the highest in vitro cumulative penetration amount of COL (235.14 ± 14.47 μg∙cm^−2^ over 48 h). Pharmacokinetic studies demonstrated that the maximum plasma drug concentration (C_max_) was 2.65 ± 0.26 ng/L and the mean retention time (MRT) was 37.47 ± 7.64 h of the COL-DIA patch, effectively reducing the drug side effects and prolonging drug activity. In addition, pharmacodynamic studies showed the patch significantly decreased the expression levels of inflammatory factors of gouty rats and reduced pathological damage in the ankle joint of rats, making it an attractive alternative to the administration of COL for the treatment of gout.

## 1. Introduction

Gout is common inflammatory arthritis characterized by monosodium urate (MSU) crystal deposition in synovial fluid, joint, and periarticular tissues. Epidemiological surveys worldwide indicate that gout prevalence is increasing, ranging from <1% to 6.8% [1]. The deposition of MSU crystals in the joints caused by chronic elevation of serum uric acid (sUA) levels above a deposition threshold is a key factor in the initiation and progression of gout [2]. The progression of gout can be defined by four pathophysiological stages: hyperuricemia, crystal deposition without symptomatic gout, crystal deposition with acute gout attacks, and advanced gout characterized by tophi, chronic gouty arthritis, and radiographic erosions [3]. In an acute gout attack, the involved joint is typically swollen, erythematous, warm, and exquisitely painful. Flares often begin overnight and remarkably adversely affect patients’ quality of life [4]. Thus, the primary purpose in the clinical treatment of acute gout attacks is to control the acute inflammatory reaction and reduce pain quickly. Various national guidelines (American College of Rheumatology, European League Against Rheumatism, and British Society for Rheumatology) recommend treatment with colchicine (COL), nonsteroidal anti-inflammatory drugs, and oral corticosteroids [5,6,7]. COL was the first drug used for gout treatment and is still the first-line drug for acute gout attacks.

COL is a tricyclic alkaloid derived from the plant Autumn Crocus. It associates with tubulin dimers to inhibit the formation of microtubules and impede cell division, thereby participating in a variety of mechanisms to regulate the inflammatory response [8,9], including inhibiting the activation of crystal-induced inflammasomes, chemotacticity of neutrophils, expression of adhesion molecules, and production of superoxides [10,11,12]. Due to its unique anti-inflammatory activity, COL is not only used for the therapy and prevention of gout, it is also used widely for familial Mediterranean fever, Behcet’s disease, and pericarditis [13,14,15]. The marketed dosage forms of COL are intended only for oral administration. The FDA banned intravenous injections in 2008 because of serious adverse effects consequences [16]. However, the bioavailability of oral COL has been reported to be about 45% and is usually accompanied by severe gastrointestinal adverse effects [17]. In addition, COL has a narrow therapeutic index because of its high cytotoxicity, with effective blood concentrations following administration ranging from 0.5 to 3 ng/mL [18,19]. Consequently, there have been cases of acute multiorgan failure and even death due to accidental overdose during therapy [20]. Thus, developing a more efficient and safe delivery system of COL is necessary and urgent.

Transdermal drug delivery is an attractive alternative to oral administration. It can bypass the first-pass effect, avoid adverse gastrointestinal side effects, maintain relatively steady drug concentrations, and allow timely termination of administration when serious adverse reactions occur [21]. Several studies have reported the feasibility of transdermal administration of COL. Most COL transdermal delivery forms are ointments [22] and gels [23,24,25,26] incorporating nanocarriers. However, dose variability and possible transfer of the drug to others from gel and ointment have emphasized the need for controlled, occluded, and safer delivery systems [27]. Therefore, transdermal patches have been developed to overcome these limitations. Joshi et al. prepared a COL-loaded solid lipid nanoparticle transdermal patch using ethyl cellulose and polyvinylpyrrolidone combined with triethyl citrate as a polymeric matrix [28]. However, on the one hand, the special storage conditions, complex production processes, and quality control required for the preparation of nanocarriers limit the application of the formulation. On the other hand, this traditional matrix-based transdermal patch suffers from poor adhesive properties and is not well suited for the joint site of gout attacks.

Studies have shown that drug-in-adhesive (DIA) patches are thinner, more comfortable, and exhibit better adhesion compared to traditional matrix-based patches [27]. In addition, the key excipient pressure-sensitive adhesives (PSAs) of DIA patches are commercially available, facilitating the production of a quality prescription product. Effective transdermal drug delivery can be made by adding skin-penetration enhancers to the patch at a low process cost. To our knowledge, no relevant studies on COL-DIA patches have been reported to date. In this study, we first screened the most suitable PSA for COL-DIA patches based on differences in the properties of different PSAs. Then, the drug content and penetration enhancers in the patch were optimized based on in vitro transdermal permeation experiments. Finally, using oral COL tablets as a control, in vivo pharmacokinetic study were performed on the optimization patch. A pharmacodynamic evaluation was also performed in a rat acute gout model using the ankle joint’s histopathological condition and the expression level of inflammatory factors as evaluation indices. The developed COL-DIA patch showed good potential for clinical application and is expected to become an alternative to oral COL for acute gout treatment.

## 2. Materials and Methods

### 2.1. Materials and Animals

Colchicine (99.41% purity) was bought from Shanxi YuNing Biotechnology Co. Ltd. (Taiyuan, Shanxi, China), and standard colchicine (99.41% purity) was bought from the Chinese Institute for Food and Drug Control (Beijing, China). Four PSAs, including Duro-Tak 87-900A (DT 900A), Duro-Tak 87-2677 (DT 2677), Duro-Tak 87-2516 (DT 2516), and Duro-Tak 87-6908 (DT 6908), were bought from Henkel AG&Co (Düsseldorf, Germany). Backing membranes (Scotch Pak^®^ 9723) and release liners (Scotch Pak^®^ 1022) were purchased from 3 M (St. Paul, MN, USA). Azone (AZ), oleic acid (OA), N-methyl pyrrolidone (NMP), propylene glycol (PG), polyethylene glycol 400 (PEG400), sodium laureth sulfate (SLA), and phenyl piperazine (PP) were purchased from the Beijing YingHua JinCheng Biotechnology Co. (Beijing, China). All other chemicals were of reagent grade and obtained from Sigma-Aldrich Sigma Aldrich Trading Co., Ltd. (Shanghai, China).

Clean-grade Sprague-Dawley rats were purchased from specific pathogen-free (SPF) Biotechnology Co. Ltd. [SCXK(Jing) 2019-0010, Beijing, China]. Prior to the investigation, they were kept for one week in SPF conditions. The Guide for the Care and Use of Laboratory Animals was followed in all animal research. All the animal experiments were reviewed and approved by the Institutional Animal Care and Use Committee of the Academy of Military Sciences (Beijing, China).

### 2.2. Solubility of COL in PSAs

When preparing COL-DIA patches using solvent evaporation, determining the maximum solubility of the drug in the PSAs is a critical step in avoiding crystallization after volatilization of the organic solvent [29]. Therefore, in this study, the maximum dissolution value of COL in each PSA (wet blend) was first determined according to a previously described method and denoted as the wet blend saturation point [30]. Then, wet mixtures of PSAs containing COL were prepared at percentages lower than the wet blend saturation point. The mixtures were cast onto glass slides and dried in an oven at 45 °C for 15 min. They were then placed at room temperature (25 ± 2 °C) for one week, and crystallization was observed under an optical microscope (ECLIPSE E100, Nikon, JAPAN). The highest percentage of COL without crystallization was chosen as the maximum solubility of COL in each PSA (based on dry weight). All concentration percentages were calculated based on the dry weight of PSAs. The formulation compositions are shown in Table 1.

### 2.3. Preparation of COL-DIA Patches

The COL-DIA patches were prepared using the solvent evaporation method. Different concentrations of COL and different types and concentrations of penetration enhancers were dissolved in ethanol using sonication. Different PSAs were added to the solution. The mixtures were stirred at 120 rpm (avoiding high shear forces) for 2 h at room temperature (25 ± 2 °C) to achieve homogeneity and then allowed to stand for 1 h to degasify. The mixtures were poured on a release liner (Scotchpak™ 1022, 3 M) and coated with a laboratory spatula coater with heating function (TBJ-X5, Nan Bei Chao, Foshan, Guangdong, China). The coating speed was 30 mm/s, and the thickness of the wet film was 300 μm. The films were kept at room temperature for 10 min and then heated for 15 min at 45 °C to remove residual organic solvents. The dried films were laminated with a backing membrane (Scotch Pak^®^ 9723, 3 M) using a roller. Finally, the obtained patches were cropped to the appropriate size for further study.

To avoid differences in thermodynamic activity caused by different solubilities, the COL-DIA patches with the maximum solubility in various PSAs were designed without any additional agent to examine the effect of different types of PSAs on transdermal effects. Results were used to select the appropriate PSA. The effect of drug loading on the skin permeation of COL was evaluated using the selected PSA. Finally, the effects of the type and concentrations of enhancers on the skin penetration of COL were investigated.

### 2.4. Determination of COL Content in the Patch

The drug content in the final optimized patches was determined as follows. The patches (1 cm^2^, *n* = 6) were immersed in 10 mL of methanol and shaken overnight. The content was transferred to a 50 mL volumetric flask and diluted with 47% methanol to the volume. The diluted solutions were centrifuged at 14,000 rpm for 10 min, and 20 μL of the supernatant was analyzed using high-performance liquid chromatography (HPLC), (1200, Agilent, Santa Clara, CA, USA). The analytical conditions of HPLC were as follows: the column was COSMOSIL C8 (4.6 × 250 mm, 5 μm). The mobile phase was 47% aqueous methanol; the detection wavelength was 254 nm; the column temperature was 25 °C; the flow rate was 1 mL/min. In addition, these patches were stored at room temperature and checked for the presence of drug crystals once a week for three months.

### 2.5. In Vitro Skin Permeation Studies

The process of transdermal absorption of a drug is that the drug is first released from the patch to the skin surface, dissolved and distributed into the lipids of the stratum corneum, diffused into the water-based active epidermis and dermis, and finally, absorbed into the body circulation by capillaries. In vitro skin permeation studies can characterize the rate and extent of transdermal drug delivery by simulating the transdermal process of the drug under physiological conditions. Skin permeability differs between different animals. Previous studies have indicated that in terms of skin permeability, pig skin is most similar to human skin except the primate skin [31]. Therefore, pig skin was used in this study. Fresh and clean pig skin was cut into a suitable size and frozen at −20 °C. The night before the experiment, the skin was thawed at room temperature. Prior to use, skins were immersed in saline solution for 30 s, and the surface water was absorbed with filter paper. The digital micrometer was used to measure the thickness of skin. Skin pieces of similar thickness were examined under a microscope to ensure the integrity of the skin barrier function. In addition, 0.9% sodium chloride solution (saline) with pH 5.5 was chosen as the receiving medium in this study because the saturation solubility of COL in saline was measured experimentally as 8.3 mg/mL, which can satisfy the leaky tank condition (the drug concentration in the receiving solution should not exceed 30% of its saturation solubility) and can well simulate the human skin microenvironment.

The effects of PSA, drug loading, and permeation enhancers on the skin permeation of COL were investigated. The permeation studies were performed using vertical Franz-type diffusion cells (TK-24Ⅱ, Shanghai Kaikai, China). The diffusion cells are a two-compartment system consisting of upper (donator) and lower chambers (acceptor), with a diffusional area of 1.77 cm^2^ and an acceptor volume of 7.5 mL. Before the experiment began, the release liners of the patches were removed, and the patches were attached to the stratum corneum side of the skin under slight pressure using a glass rod. The skin with the patch attached was sandwiched between the donator and acceptor chambers, with the dermal layer in contact with the acceptor chamber. Saline solution was added to the acceptor chamber equipped with a magnetic stirrer. The rotating speed was set to 200 rpm, and the water bath temperature was set to 37 °C, providing a skin surface temperature of 32 °C. Subsequently, samples (1 mL) were drawn from the acceptor chamber at fixed time points (2, 4, 6, 8, 10, 12, 24, 30, 36, and 48 h) and immediately replaced with an equal volume of isothermal fresh saline. The samples were centrifuged at 14,000 rpm for 5 min, and the supernatants were collected. Supernatants (20 μL) were analyzed using HPLC, and the peak area was recorded for calculating the cumulative drug skin penetration using the following formula:

$$Q=C_{n}\times V+\overset{n-1}{\underset{i=1}{\sum }}C_{i}\times V_{i}/A$$

where Q is the cumulative amount of permeated COL per unit area; $C_{n}$ and $C_{i}$ are the drug concentrations of the nth and ith samples, respectively; V represents the volume of the acceptor (7.5 mL); $V_{i}$ represents the sampling volume (1 mL), and A represents the effective diffusion area (1.77 cm^2^). The cumulative permeated drug amount Q was plotted against time, and the steady-state flux (Jss, μg∙cm^−2^∙h^−1^) was calculated from the slope of the linear part of the curve.

### 2.6. In Vivo Pharmacokinetic Studies

#### 2.6.1. In Vivo Pharmacokinetic Experimental Design

The final optimized COL-DIA patches from in vitro studies were applied to rat dorsal skin to determine the pharmacokinetic profile of transdermally administered COL-DIA patches compared with orally administered COL. A total of 12 healthy Sprague–Dawley male rats (240–260 g) were randomly divided into two groups (6 rats/group): COL-Ts (oral tablet group) and COL-TDDs (transdermal patch group). For 12 h before the experiment, rats in the COL-Ts group were fasted but allowed to drink water freely, and rats in the COL-TDDs group had dorsal hair removed using an animal razor, while the skin was carefully examined for any scratches or damage.

COL tablets were dissolved in saline for oral administration to rats (0.6 mg/kg). For transdermal administration to rats, 4 cm^2^ patches were applied to the dorsal skin of rats (equivalent to an oral COL dose of 6.4 mg/kg). Rats were placed in separate small cages after drug administration. Blood samples (0.5 mL) were collected from the orbital venous plexus of rats at time points of 0, 0.08, 0.5, 1, 2, 4, 6, 8, 12, 24 h after oral administration and 0, 1, 2, 3, 4, 6, 8, 12, 24, 36, 48 h after patch administration. Blood samples were collected in sodium heparin tubes, immediately centrifuged at 3000 rpm for 15 min, and stored at −20 °C until analysis.

#### 2.6.2. Determination of Drug Concentration in Plasma

The plasma samples were extracted using solid-phase extraction. Plasma samples (100 μL) were placed in 1.5 mL Eppendorf tubes with 10 μL of the internal standard (10 ng/mL verapamil hydrochloride), and 900 μL of mixed organic solvent (methyl tert-butyl ether: ethyl acetate = 1:9, *v*/*v*) were added. The mixture was vortexed for 3 min and centrifuged at 14,000 rpm for 10 min. Then, the upper organic layer was transferred into 2 mL centrifuge tubes and evaporated to dryness at 50 °C using a concentrating centrifuge (Cleanert, Agela Technologies, Wilmington, DE, USA). The residue was reconstituted in 40% acetonitrile (100 μL), vortexed for 3 min, and centrifuged at 14,000 rpm for 10 min. Finally, 5 µL of the supernatant was injected into an Agilent HPLC-electrospray ionization-tandem mass spectrometry system (HPLC-MS/MS).

The concentration of COL in the rat plasma was determined using HPLC–MS/MS. This system consisted of an Agilent 1200 series HPLC coupled to an Agilent 6410 triple quadrupole mass spectrometer equipped with an electrospray ionization (ESI) source (Agilent Technologies, Santa Clara, CA, USA). Chromatographic separation was carried out at 40 °C using a C18 reversed-phase column (Agilent narrow Bore RR SB-C18, 2.1 × 100 mm, 3.5 m, Agilent Inc., USA). Acetonitrile and 10 mM ammonium acetate with 0.1% (*v*/*v*) formic acid (40:60, *v*/*v*), made up the mobile phase. A flow rate of 0.3 mL/min was used. For quantitative analysis, the MS was run in the multiple reaction monitoring (MRM) mode under negative ionization. The MRM transitions for COL were 400.1 → 358.1 (m/z) and for verapamil hydrochloride, they were 455.2 → 165.1 (m/z).

The following optimized MS settings were used: collision energy of 21 eV and 30 eV for COL and verapamil hydrochloride, respectively; fragment voltage of 140 V and 160 V; capillary voltage of 4000 V; gas temperature of 350 °C; gas flow of 12 L/min; and nebulizer pressure of 35 psi.

### 2.7. In Vivo Pharmacodynamics Studies

#### 2.7.1. Establishment of an Acute Gouty Arthritis Model and Treatment Scheme

Healthy male Sprague–Dawley rats (240–260 g) were randomly segregated into four groups (*n* = 12 per group): control (Con), MSU model (MSU), COL tablet-treated (Col-Ts), and COL-DIA patch-treated (Col-TDDs). Except for the Con group, the rats in the other groups were injected 0.2 mL of MSU suspension (2.5 g/100 mL) in the articular cavity between the median ankle joint and the tibiofibular using a sterile syringe. The rats in the Con group were injected with an equivalent volume of physiological saline at the same site. One hour after modeling, rats in the Col-Ts group were administered COL tablet solution (0.6 mg/kg) by gavage once a day until the end of the experiment (48 h after modeling). For the Col-TDDs group, a 4 cm^2^ in vitro optimal patch was applied to the skin of the rat ankle joint and secured with medical tape. At the end of the experiment, the patch was removed.

#### 2.7.2. Observation Indicator and Curative Effect Evaluation

The left ankle circumference of the rats was measured at the same position before modeling and at 6, 12, 24, and 48 h after modeling using a non-elastic cotton thread. The swelling index (%) was calculated according to the following formula: swelling index (%) = [(ankle circumference after modeling − ankle circumference before modeling)/ankle circumference before modeling] × 100%.

Twenty-four hours after modeling, six rats were randomly selected from each group. The rats were anesthetized using isoflurane. First, the left ankle joint of the rats was lavaged to collect joint lavage fluid. Centrifuging the collected lavage fluid at 10,000 rpm for 10 min. Until analysis, the supernatants were collected and kept at −80 °C. The rats were then sacrificed, and whole blood was collected from the abdominal aorta. The collected blood samples were allowed to stand for 30 min at room temperature to clot, then centrifuged at 3000 rpm for 10 min. The supernatant (serum) was removed and stored at −80 °C until analysis. The modeled ankle joints were then removed, fixed for 48 h in 4% paraformaldehyde solution, decalcified for 20 days in 10% ethylenediaminetetraacetic acid solution, dehydrated in gradient ethanol, and then embedded in paraffin. Using a microtome, the tissues were cut into 5 µm-thick sections, which were then heated to 65 °C for 1 h. The sections were then stained with hematoxylin and eosin (HE), and histopathological changes in the synovial tissue were observed under a microscope. The levels of pro-inflammatory cytokines Tumour necrosis factor-α (TNF-α) and Interleukin-1β (IL-1β) in the joint lavage fluid and serum were measured using commercial ELISA kits (Solarbio, Beijing, China). The same procedure was performed on the remaining six rats in each group 48 h after modeling.

## 3. Results and Discussion

### 3.1. Solubility of COL in PSAs

This study investigated four PSAs: acrylate copolymers with different functional groups (DT 900A, DT 2677, DT 2516) and polyisobutylene (DT 6908). Through visual observation of the wet blends, it was found that COL’s wet blend saturation points in DT 900A, DT 2677, and DT 2516 were 2.33%, 11.39%, and 15.66%, respectively. However, COL was almost insoluble in DT 6908, and the wet blend saturation point was less than 1%. Therefore, no further research was done on DT 6908. As a further step, the maximum solubility of COL in the three PSAs was determined by slide crystallization experiments. Slide crystallization is a relatively fast tool used as an alternative to preparing a complete patch because it can mimic the nature of the final cast laminate [32,33]. Slide crystallization data are shown in Table 1. The results indicate that the maximum solubilities of COL in DT 900A, DT 2677, and DT 2516 were 1.50%, 10.00%, and 12.00%, respectively. All concentration values were calculated based on the dry weights of the PSAs.

### 3.2. Preparation of COL-DIA Patches

#### 3.2.1. Effect of PSAs on the Skin Permeation of COL

**Figure 1:**
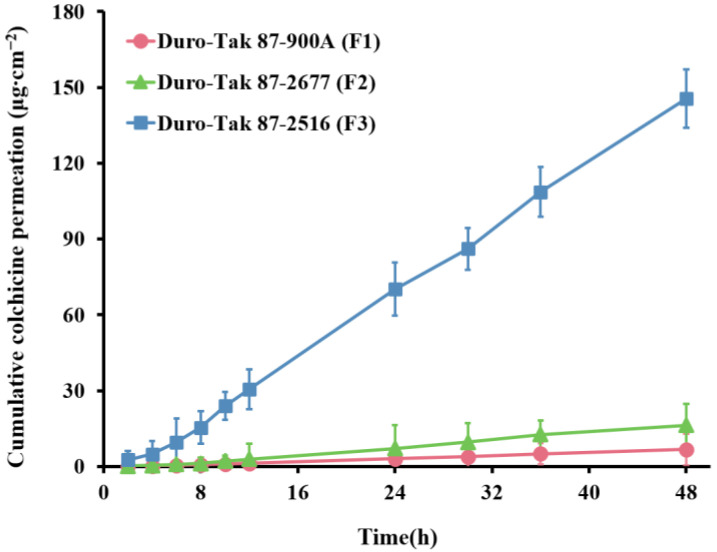
In vitro pig skin permeation profiles of colchicine in patches containing colchicine with highest solubility in various PSAs (mean ± SD, *n* = 6).

The selection of PSAs is the primary and critical step in the TDDs formulation design [34]. The differences in the solubility of the drug in PSAs and the nature of the PSA itself (e.g., structure, intrinsic thermodynamic activity, and drug–PSA interaction) affect the permeation flux [35,36]. Maximum thermodynamic activity is obtained when the drug is at its highest solubility level, allowing for the greatest permeation flux [32,34]. Therefore, to select the appropriate PSA, DIA patches with the highest solubility of COL were designed to evaluate the effect of different properties of PSA on skin permeability of COL. The transdermal permeation curves of COL for the three PSAs are shown in Figure 1, and the formulation composition and permeation parameters are listed in Table 2.

Table 2 indicates that there was a significant difference in the cumulative amounts of COL permeants in the three PSAs, with the highest cumulative permeation in DT 2516, followed by DT 2677 and DT 900A. It is undeniable that although all were at the highest concentration levels, the concentration gradient provided by the drug loadings was one of the reasons for this difference. However, this was not the most important factor. Notably, although the percentage of drug loading of DT 2677 was 6.7 times that of DT 900A, its cumulative permeation was only 2.4 times that of DT 900A, and there was no significant difference in the permeation flux between the two. It is speculated that this may be because DT 900A is a non-functional PSA, while DT 2677 PSA contains carboxy-functional groups, which may have strong interactions with alkaline drug COL and hinder the release of drug molecules [35,37]. In addition, the intrinsic thermodynamic activity of PSAs is an important factor affecting skin permeability. DT 2677 and DT 2516 have a glass transition temperature of −25 °C and −43 °C, respectively. The glass transition temperature is a simple indicator of the intrinsic thermodynamic activity of PSAs [35,36]. The lower glass transition temperature of DT 2516 indicates more free volume formation, which is more favorable for drug diffusion. This suggests that PSA plays an integral role in the in vitro permeation flux of drugs.

Ultimately, in this study, DT 2516 was selected as the PSA for COL-DIA patches because of its obvious advantages, namely, the highest cumulative permeation and permeation flux.

#### 3.2.2. Effect of COL Loading on Skin Permeation

**Figure 2:**
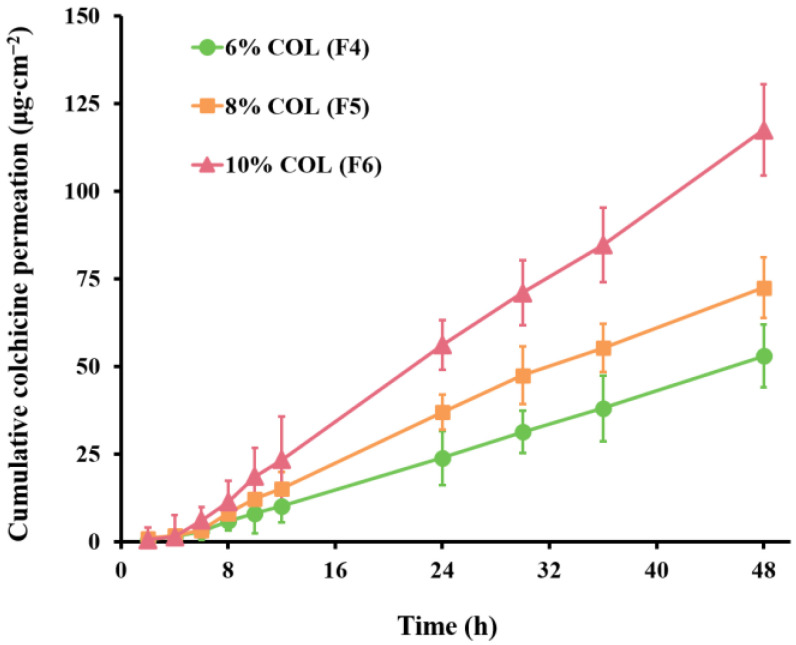
In vitro pig skin permeation profiles of colchicine (COL) from patches containing different contents of COL (*w*/*w*%, based on PSAs dry weight) in Duro-Tak 87-2516 (mean ± SD, *n* = 6).

Drug content is considered to be one of the important characteristics of TDDs. The drug content in the transdermal patches is often higher than the delivered dose during use to achieve clinically effective delivery rates. Because the concentration of the active ingredient may be close to its saturation limit, there is a risk of crystallization of the drug during storage of the product, with possible adverse effects on the quality and efficacy of the product. In this study, the effect of drug content (8.00, 9.00, 10.00, and 12.00%) on the skin penetration of COL was further evaluated using DT 2516-based patches. Figure 2 shows the skin permeation profiles for different COL contents. The composition and permeation parameters of the formulations are listed in Table 3.

During the experiments, crystallization was observed at 12.00% drug content of COL-DIA patches after 2 months at room temperature, suggesting that slide crystallization can only be used to estimate the maximum solubility of the drug in the PSA. The actual patch thickness, processing conditions, and amplification procedures may affect the crystallization [38]. In addition, since the relatively low diffusion coefficient of the drug in this high viscosity system and the need for nucleation for crystallization initiation, so crystallization may not occur immediately after production [39]. Finally, the drug content for which no crystals were observed (8.00, 9.00, 10.00%) was selected for further study. As Table 3 indicates, the permeation flux of COL increased with an increase of drug content in the patches. This was because the range of drug content studied was below the saturation threshold. According to Fick’s law of diffusion, when the drug concentration in a patch is below its saturation solubility, the amount of skin penetration of the drug is proportional to its concentration. Notably, when the COL content of the patch was 10.00% (*w*/*w*%, based on PSAs dry weight), corresponding to a permeation flux of 2.44 ± 0.32 μg∙cm^−2^∙h^−1^, this was still higher than the permeation flux (0.37 ± 0.06 μg∙cm^−2^∙h^−1^) at the maximum solubility level (10%) of DT 2677-based. This further illustrates the importance of the PSA performance and feasibility of using DT 2516 as the optimum PSA. The final COL content of the patch was 10.00%.

#### 3.2.3. Effect of Permeation Enhancers on the Skin Permeation of COL

**Figure 3:**
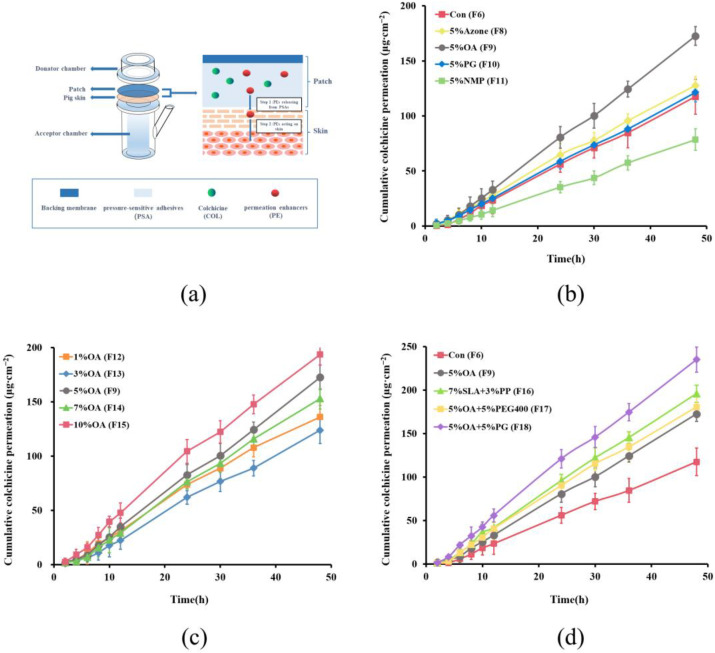
(**a**) The schematic diagram of screening device of the permeation enhancers and its action on the skin. In vitro pig skin permeation profiles of colchicine from patches containing 10% drug with (**b**) different single permeation enhancers, (**c**) different concentrations of oleic acid (OA), (**d**) binary permeation enhancers in Duro-Tak 87-2516 (mean ± SD, *n* = 6). All the concentration values were calculated (*w*/*w*%) based on the dry weight of PSAs.

Multiple strategies have been developed to improve transdermal drug penetration by overcoming the intrinsic barrier of the stratum corneum [21]. The use of chemical permeation enhancers (CPEs) is the preferred and most commonly used approach. The mechanism by which CPEs enhance drug permeation relies on their influence on skin barrier structure and formulation properties [40,41]. In this study, the effect of permeation enhancers on the skin permeability of COL was assessed by preparing DT 2516-based patches containing different types and concentrations of single enhancers (AZ, OA, PG, and NMP) and binary permeation promoters (SLA+PP, OA+PEG 400, and OA+PG). The concentration of COL was set to 10% (*w*/*w*%) based on the percentage of the dry weight of PSA. Figure 3 shows the permeation profiles of COL in different patches. The corresponding formulation compositions and relevant permeation parameters are listed in Table 4.

As shown in Table 4, compared with patches without penetration enhancers (control), the permeation flux of COL in patches containing AZ, OA, and PG increased to different degrees, whereas NMP slightly decreased the permeation of COL. OA showed the most significant effect, increasing the permeation flux of COL 1.47-fold compared to the control. Therefore, OA was selected for further studies. By examining the permeation-enhancing effects of OA at different concentrations, we found that 10% OA had the most significant effect, increasing the permeation flux of COL 1.69-fold compared to the control. However, single higher concentrations of CPEs tend to achieve strong enhancement along with skin irritation, while the combined application of CPEs may achieve more satisfactory efficacy and safety owing to synergistic effects [42]. Therefore, in this study, binary permeation enhancer combinations (7% SLA + 3% PP, 5% OA + 5% PG, and 5% OA + 5% PEG) were chosen to investigate their effect on COL skin permeability further. The results showed that the combination of OA and PG significantly promoted the permeation of COL, increasing the permeation flux to 2.01-fold of the control. This may be because PG can solubilize keratin in the stratum corneum, and OA can disturb skin lipids, reducing the barrier effect of the stratum corneum. PG and OA can synergize by improving each other’s skin permeability, resulting in an overall enhancing effect [43,44]. In addition, PG may interfere with the hydrogen bonding between OA and PSA, reducing their interaction and thus improving the release of OA from the patches [45,46]. Finally, we selected 5% OA + 5% PG as the COL-DIA patch permeation enhancer.

Formulation (F18) containing 10% COL, 5% OA, 5% PG, and DT 2516 as PSA was selected as the final formulation of the COL-DIA patch to obtain maximum COL delivery. The colchicine content in this formulation was determined to be 401.35 ± 8.15 μg/cm^2^. At 25 ± 2 °C and 60% ± 10% RH, no drug crystallization was observed in the patch under the microscope for three months, and there was no significant change in the drug content of the patch. The results showed that the patch we prepared has good stability. This formulation was used in subsequent in vivo studies.

### 3.3. In Vivo Pharmacokinetic Studies

**Figure 4:**
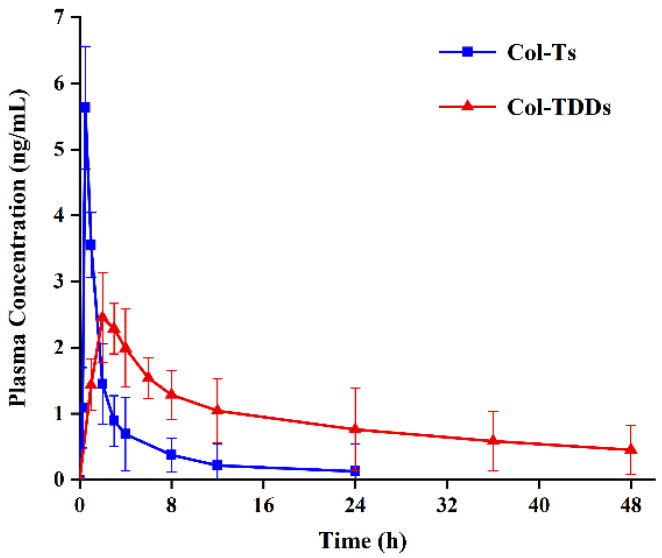
Plasma concentration-time profiles of colchicine in rats after transdermal patch (6.4 mg/kg) and oral (0.6 mg/kg) administration in a single-dose study (mean ± SD, *n* = 6).

The mean plasma drug concentration versus time profiles in rats after single-dose oral administration of COL tablet solution and transdermal administration of COL-DIA patch are shown in Figure 4. The pharmacokinetic parameters are shown in Table 5. After oral administration (0.6 mg/kg), the blood concentration of COL peaked rapidly and then decreased sharply with a T_max_ of 0.5 h, whereas after transdermal administration (6.4 mg/kg, 1.6 mg/4 cm^2^), the T_max_ in plasma was approximately at 2.5 h, and a persistent and stable plasma drug concentration was provided over 48 h. There was a significant difference in C_max_ between the two modes of administration (2.65 ± 0.26 ng/mL for transdermal administration vs. 5.38 ± 1.07 ng/mL for oral administration). Compared to the mean residence time (MRT) of 4.92 ± 1.25 h for oral administration, the MRT was significantly longer in the transdermal administration group (MRT = 37.47 ± 7.64 h). Besides, the relative bioavailability of colchicine after transdermal administration of the optimal patches is 46.69%, based on the (AUC_transdermal plasma_ × oral dose)/(AUC_oral plasma_ × transdermal dose) × 100%. COL has been reported to have a narrow therapeutic window due to high cytotoxicity. Effective steady-state concentration in the blood ranging from 0.5 to 3 ng/mL following treatment of acute gout, with approximately 3 ng/mL usually causing toxic reactions [19]. The most common adverse reactions, such as nausea, vomiting, diarrhea and other gastrointestinal reactions, are dose-dependent [47]. Compared with COL tablets, the COL-DIA patch avoids the blood concentration peak-and-trough phenomenon, which could reduce drug toxicities while achieving effective therapeutic concentrations. Additionally, its relatively long MRT could maintain effective blood concentration and provide controlled and prolonged drug activity.

### 3.4. In Vivo Pharmacodynamics Studies

**Figure 5:**
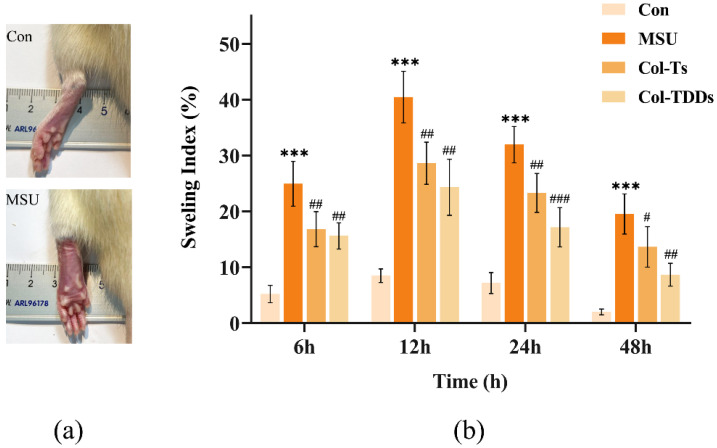
(**a**) Pictures of the ankle joints of rats in Con and MSU groups 12 h after injection of MSU suspension. (**b**) Effect of colchicine drug-in-adhesive patches and colchicine tablets on swelling index of the ankle in MSU-induced gout rats. Data are expressed as the mean ± SD; *** *p* < 0.001 vs. Con. ^#^ *p* < 0.05, ^##^ *p* < 0.01, ^###^ *p* < 0.001 vs. MSU.

MSU crystals, the causative agent of gout, are a potent pro-inflammatory stimulus. Phagocytosis of MSU crystals stimulates macrophages residing in the joints, triggering the release of many inflammatory factors, such as IL-1β and TNF-α, and the infiltration of large numbers of neutrophils into inflamed joints [2,48]. The present study examined the effect of COL-DIA patches on the degree of joint swelling in rats with MSU-induced gout. Compared with the Con group, the left ankle joints of the rats in the MSU group exhibited severe redness and swelling after the injection of the MSU solution, with the maximum swelling occurring after 12 h (Figure 5a). Subsequently, the redness and swelling of rat joints in all groups gradually improved. Figure 5b shows the joint swelling index of each group at different time points. At each time point, the ankle swelling index was significantly higher in the MSU group than in the Con group. Meanwhile, rats in the Col-Ts and Col-TDDs groups showed significant inhibition of MSU-induced ankle swelling following drug intervention.

**Figure 6:**
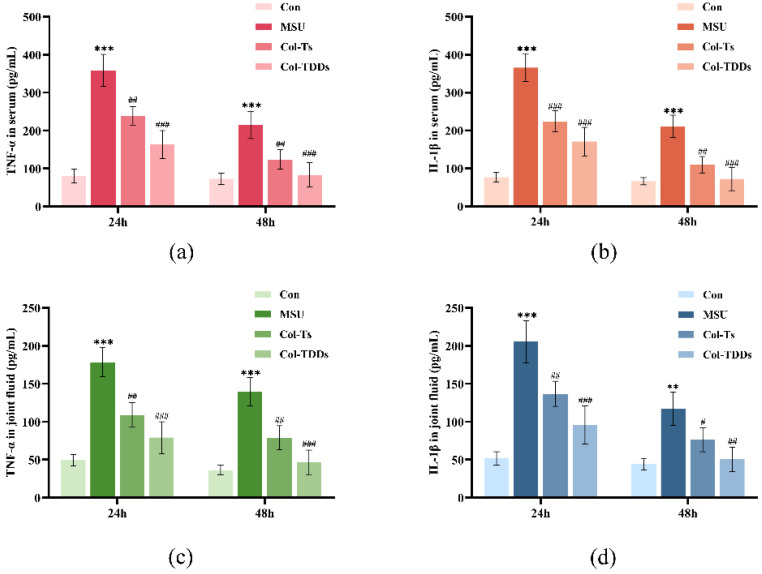
Effect of colchicine drug-in-adhesive patches and colchicine tablets on the levels of key inflammatory factors (**a**) TNF-α in rat serum and (**b**) IL-1β in rat serum, and (**c**) TNF-α in rat joint fluid and (**d**) IL-1β in rat joint fluid. Data are expressed as the mean ± SD; ** *p* < 0.01, *** *p* < 0.001 vs. Con. ^#^ *p* < 0.05, ^##^ *p* < 0.01, ^###^ *p* < 0.001 vs. MSU.

Next, the effects of the COL-DIA patch on the TNF-α and IL-1β levels in rat serum and joint fluid were examined. The ELISA results (Figure 6) showed that the expression of IL-1β and TNF-α in both serum and joint fluid were significantly upregulated after MSU injection. At 24 h, the expression of inflammatory factors was upregulated 3- to 5-fold in the MSU group compared with the Con group, indicating intense inflammation. Compared to the MSU group, the expression of inflammatory factors was substantially decreased in the Col-Ts and Col-TDDs groups, probably because of the powerful anti-inflammatory activity of COL, which could block the release of IL-1β and TNF-α by inhibiting the activation of NLRP3 inflammasome and Caspase 1. Although the expression levels of these inflammatory factors induced by MSU were gradually suppressed by autoimmunity over time, especially 48 h after modeling, there were still significant differences in the expression levels of IL-1β and TNF-α between the model and treatment groups. Notably, the Col-TDDs group exhibited a higher inhibition of inflammation than the Col-Ts group. This could be attributed to the direct action of the COL-DIA patch on the lesion and the sustained and slow release of the drug from the patch.

**Figure 7:**
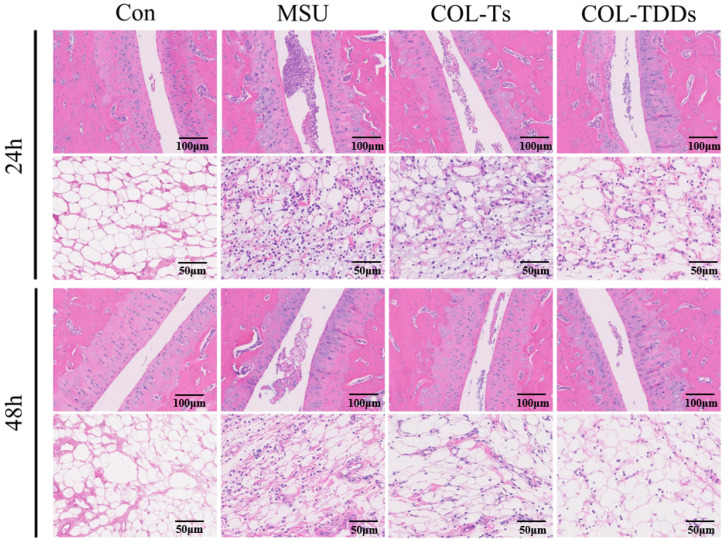
Histopathological study of ankle joints obtained 24 h and 48 h post-MSU injection.

Finally, the effect of COL-DIA patches on MSU crystal-induced ankle histological manifestations was examined. A large number of neutrophils infiltrating inflamed joints is the main feature of acute gouty arthritis. As shown in Figure 7, the ankle joints of rats in the Con group were normal and there was no significant inflammatory infiltration. In contrast, the rats in the MSU group showed thickened articular cartilage and a lot of inflammatory cells infiltrating the narrow articular cavity. Symptoms improved 48 h after MSU injection, but notable inflammatory cell infiltration was still observed in the MSU group rats. Compared to the MSU group, the pathological damage in the ankle joint of experimental rats treated with COL was significantly attenuated, presumably because COL could inhibit the contact between MSU crystals and monocytes and inhibit leukocyte accumulation in the ankle joint. Notably, the inflammatory condition of rats in the COL-TDDs group showed a more significant improvement compared to the Col-Ts group, which may due to the relatively long MRT of COL-DIA patches and the ability to act directly on the lesion site.

## 4. Conclusions

In this study, COL-DIA patches were prepared and optimized by in vitro permeation studies using different PSA, drug loading, and permeation enhancers. The optimized patch formulation, containing 10% COL, 5% OA, and 5% PG in Duro-Tak 87-2516, was selected for in vivo studies. Compared with oral administration, the distribution of COL after transdermal administration showed stable plasma COL levels and sustained release in vivo. Furthermore, positive efficacy of the patch was observed in rats with acute gouty arthritis. These results suggest that COL-DIA patches are an attractive alternative COL formulation to treat acute gout.

## Figures and Tables

**Table 1:** Formulations prepared to determine the solubility of colchicine in various PSAs and the results of slide crystallization.

Pressure-Sensitive Adhesives (PSAs)	Functional Groups	Contents (mg)			Drug (% *w*/*w*) ^1^	Crystallization
		Adhesive Wet Weight	Adhesive Dry Weight	Amount of Drug		
Acrylate Duro-Tak 87-900A	none	2000	860	4.30	0.50	No
		2000	860	8.60	1.00	No
		2000	860	12.90	1.50	No
		2000	860	17.20	2.00	Yes
Acrylate Duro-Tak 87-2677	-COOH	2000	790	63.20	8.00	No
		2000	790	71.10	9.00	No
		2000	790	79.00	10.00	No
		2000	790	86.90	11.00	Yes
Acrylate Duro-Tak 87-2516	-OH	2000	830	66.40	8.00	No
		2000	830	83.00	10.00	No
		2000	830	99.60	12.00	No
		2000	830	124.50	15.00	Yes
PIB Duro-Tak 87-6908	none	2000	760	The wet blend saturation point was less than 1%, so further research was discontinued		
		2000	760			
		2000	760			
		2000	760			

^1^ All the concentration values were calculated based on the dry weight of PSAs.

**Table 2:** In vitro pig skin permeation parameters of colchicine in patches containing colchicine with highest solubility in various PSAs (mean ± SD, *n* = 6).

Code	Formulation Details			Q_48h_ (μg∙cm^−2^)	Flux (μg∙cm^−2^∙h^−1^)	T_lag_ (h)
	PSAs	Colchicine Loading	Enhancers			
F1	Duro-Tak 87-900A	1.50%	-	6.68 ± 3.39 **	0.15 ± 0.11 **	3.25 ± 0.42 **
F2	Duro-Tak 87-2677	10.00%	-	16.29 ± 4.61 **	0.37 ± 0.06 **	3.57 ± 0.51 **
F3	Duro-Tak 87-2516	12.00%	-	146.54 ± 8.52	3.15 ± 0.23	1.83 ± 0.18

PSAs pressure-sensitive adhesives, Q_48h_ cumulative colchicine permeation at 48 h, T_lag_ lag time, SD standard deviation. ** Significant differences from F3 (Duro-Tak 87-2516) at *p* < 0.01.

**Table 3:** In vitro pig skin permeation parameters of colchicine from patches containing different contents of colchicine (*w*/*w*%, based on PSAs dry weight) in Duro-Tak 87-2516 (mean ± SD, *n* = 6).

Code	Formulation Details			Q_48h_ (μg∙cm^−2^)	Flux (μg∙cm^−2^∙h^−1^)	T_lag_ (h)
	PSAs	Colchicine Loading	Enhancers			
F4	Duro-Tak 87-2516	6.00%	-	52.95 ± 9.05 **	1.13 ± 0.12 **	1.92 ± 0.19
F5	Duro-Tak 87-2516	8.00%	-	72.44 ± 8.75 **	1.64 ± 0.14 **	1.81 ± 0.33
F6	Duro-Tak 87-2516	10.00%	-	117.44 ± 12.98	2.44 ± 0.32	1.96 ± 0.21
F7	Duro-Tak 87-2516	12.00%	-	Crystallization after two months at room temperature		

PSAs pressure-sensitive adhesives, Q_48h_ cumulative colchicine permeation at 48 h, T_lag_ lag time, SD standard deviation. ** Significant differences from F6 (colchicine loading 10%) at *p* < 0.01.

**Table 4:** In vitro pig skin permeation parameters of colchicine from patches containing 10% colchicine and different permeation enhancers in Duro-Tak 87-2516 (mean ± SD, *n* = 6).

Code	Formulation Details			Q_48h_ (μg∙cm^−2^)	Flux (μg∙cm^−2^∙h^−1^)	T_lag_ (h)
	PSAs	Colchicine Loading	Enhancers			
F6	Duro-Tak 87-2516	10.00%	-	117.36 ± 12.98	2.44 ± 0.32	1.96 ± 0.21
F8	Duro-Tak 87-2516	10.00%	5%AZ	127.68 ± 8.31	2.72 ± 0.41	1.69 ± 0.17
F9	Duro-Tak 87-2516	10.00%	5%OA	172.62 ± 8.48 **	3.59 ± 0.17 **	0.89 ± 0.33 **
F10	Duro-Tak 87-2516	10.00%	5%PG	120.26 ± 8.47	2.55 ± 0.61	1.92 ± 0.15
F11	Duro-Tak 87-2516	10.00%	5%NMP	78.50 ± 9.76 **	1.68 ± 0.39 *	2.94 ± 0.39 **
F12	Duro-Tak 87-2516	10.00%	1%OA	139.94 ± 10.76 *	3.05 ± 0.28 *	1.38 ± 0.20 *
F13	Duro-Tak 87-2516	10.00%	3%OA	123.91 ± 12.31	2.71 ± 0.56	1.91 ± 0.37
F14	Duro-Tak 87-2516	10.00%	7%OA	152.97 ± 9.47 **	3.34 ± 0.12 **	0.95 ± 0.36 **
F15	Duro-Tak 87-2516	10.00%	10%OA	193.65 ± 9.98 **	4.14 ± 0.24 **	0.82 ± 0.24 **
F16	Duro-Tak 87-2516	10.00%	7%SLA + 3%PP	195.86 ± 9.64 **	4.27 ± 0.31 **	1.82 ± 0.15
F17	Duro-Tak 87-2516	10.00%	5%OA + 5%PEG	180.65 ± 12.17 **	3.96 ± 0.18 **	1.84 ± 0.21
F18	Duro-Tak 87-2516	10.00%	5%OA + 5%PG	235.14 ± 14.47 **	4.91 ± 0.22 **	1.02 ± 0.18 **

PSAs pressure-sensitive adhesives, AZ Azone, OA oleic acid, NMP N-methyl pyrrolidone, SLA sodium laureth sulfate, PP phenyl piperazine, PG propylene glycol, PEG400 polyethylene glycol 400, Q_48h_ cumulative colchicine permeation at 48 h, T_lag_ lag time, SD standard deviation. All the concentration values were calculated based on the dry weight of PSAs. * Significant differences from F6 (Control without penetration enhancers) at *p* < 0.05. ** Significant differences from F6 (Control) at *p* < 0.01.

**Table 5:** Pharmacokinetic parameters of colchicine in plasma following transdermal (6.4 mg/kg) and oral (0.6 mg/kg) administration in rats (mean ± SD, *n* = 6).

Parameters	Oral Administration	Transdermal Administration
C_max_ (ng/L)	5.38 ± 1.07	2.65 ± 0.26
T_max_ (h)	0.50 ± 0.00	2.25 ± 0.50
MRT (h)	4.92 ± 1.25	37.47 ± 7.64
AUC_0–∞_ (ng/L·h)	12.66 ± 1.55	63.06 ± 6.93

C_max_, maximum concentration; T_max_, time of maximum concentration; MRT, the mean residence time; AUC_0–∞_, area under the plasma concentration-time curve.

## Data Availability

Not applicable.
